# Neferine Exerts Ferroptosis-Inducing Effect and Antitumor Effect on Thyroid Cancer through Nrf2/HO-1/NQO1 Inhibition

**DOI:** 10.1155/2022/7933775

**Published:** 2022-06-27

**Authors:** Shujing Li, Yanyan Zhang, Jin Zhang, Bo Yu, Weiran Wang, Baosong Jia, Jingtao Chang, Jing Liu

**Affiliations:** ^1^Department of Thyroid Surgery, The First Hospital of Shanxi Medical University, Taiyuan, Shanxi 030001, China; ^2^Department of General Surgery, The First Hospital of Shanxi Medical University, Taiyuan, Shanxi 030001, China; ^3^Department of General Surgery, Shanxi Medical University, Taiyuan, Shanxi 030001, China

## Abstract

Thyroid cancer is the most prevalent endocrine malignancy with an increasing incidence in the past few decades. Neferine possesses various pharmacological activities, which have been applied in diverse disease models, including various tumors. However, the detailed effect and mechanism of neferine on thyroid cancer are still unclear. In the current study, the viability of IHH-4 and CAL-62 cells was examined by the CCK-8 assay. The effect of neferine on the proliferation, apoptosis, invasion, vascular endothelial growth factor (VEGF), epithelial-mesenchymal transition (EMT), and ferroptosis was evaluated by CCK-8, flow cytometry, western blot, and spectrophotometry assays. Mechanically, the expressions levels of Nrf2/HO-1/NQO1 signaling were first determined by a western blot, which was then verified by Nrf2 overexpression. *In vivo* validation was also conducted on BALB/*c* nude mice with an inoculation dose of 2 × 10^6^ IHH-4 cells. The results showed that neferine repressed the viability of both IHH-4 and CAL-62 cells both in a dose-dependent way and in a time-dependent fashion, in which the IC50 value of neferine on IHH-4 and CAL-62 cells was 9.47 and 8.72 *μ*M, respectively. Besides, neferine enhanced apoptosis but suppressed invasion, angiogenesis, and EMT of IHH-4 and CAL-62 cells. Moreover, neferine induced the activation of ferroptosis in thyroid cancer cells. Notably, it was revealed that the Nrf2/HO-1/NQO1 pathway was strongly associated with the effect of neferine on the modulation of thyroid cancer. Furthermore, these outcomes were validated in xenografted mice. Therefore, neferine exerted an antitumor effect and ferroptosis-inducing effect on thyroid cancer via inhibiting the Nrf2/HO-1/NQO1 pathway.

## 1. Introduction

Thyroid cancer is the most prevalent endocrine malignancy, whose incidence has rapidly increased in the past few decades [1]. It mainly comprises three histological types, including undifferentiated, differentiated, and medullary thyroid cancer [2]. Among them, the differentiated thyroid cancer contains follicular, and papillary thyroid cancer exhibits an excellent prognosis with 80–95% 10-year survival rates, while undifferentiated thyroid cancer designates anaplastic, and poorly differentiated thyroid cancer has an extraordinary poor survival with prominent invasiveness [3, 4]. Moreover, both the differentiated and undifferentiated thyroid cancer forms show distant metastasis [5, 6]. Currently, several therapies have been applied in the treatment of thyroid cancer, such as radioactive iodine therapy, surgery, thyroid-stimulating hormone (TSH) as well as immunotherapeutic agents [7, 8]. Despite the encouraging advance, much exploration remains to contribute to the understanding and development of pathogenesis and treatment of thyroid cancer.

Ferroptosis distinguished from both apoptosis and necrosis that is a fresh form of cell death featured with iron-induced lipid peroxidation [9]. Although ferroptosis can be activated in a variety of pathological states, such as lung injury, neurodegeneration, and renal failure [10, 11], tumor cells can adapt to the oxidative environment to confine ferroptosis, thereby remodeling the tumor niche to facilitate cancer growth and development [12]. Thus, ferroptosis activation is a potential option for the treatment of diverse cancers, including thyroid cancer. Wang et al. [13] reports that E26 transformation specific (ETS) variant 4 (ETV4), an ETS family transcription factor, can suppress ferroptosis, which facilitates the progression of papillary thyroid cancer. Consistently, the suppressive effect of vitamin C on anaplastic thyroid cancer is also demonstrated to be through the induction of ferroptosis [14]. In addition, Chen et al. [15] shows that circKIF4A inhibits ferroptosis to promote the malignant development of papillary thyroid cancer via the miR-1231/GPX4 axis.

Neferine is one of the most abundant bisbenzylisoquinoline alkaloids extracted from the seed embryos of lotus [16]. Eviden+ces have revealed that neferine owns various pharmacological activities, including anti-inflammation, antioxidation, antiarrhythmia, antihypertension, antithrombosis, antiplatelet, and antiamnesia; thus, it has been employed for the treatment of high fevers, arrhythmia, hypertension, occlusion, platelet aggregation, obesity, and hyposomnia [16]. Moreover, a growing number of studies have illustrated that neferine has a therapeutic effect on various cancers. For instance, neferine impedes growth and mobility of human prostate cancer stem cells via activating the p38 MAPK/JNK pathway [17]. Neferine restricts lung carcinogenesis induced by diethylnitrosamine in Wistar rats [18]. Neferine is a powerful anticancer agent against SiHa and HeLa cervical cancer cells by the induction of autophagy and apoptosis associated with reactive oxygen species (ROS) [19]. Nevertheless, the detailed effect and mechanism of neferine on thyroid cancer remain intricate.

Therefore, the current study aimed to discuss the detailed effect and mechanism of neferine on thyroid cancer. We first discussed the effect of neferine on the growth, invasion, and epithelial-mesenchymal transition (EMT) of thyroid cancer cell lines, IHH-4, and CAL-62. Then, the role of neferine in the ferroptosis was investigated in IHH-4 and CAL-62 cells. Moreover, the underlying mechanism was also explored through the examination of the expressions involved in the Nrf2/HO-1/NQO1 signaling pathway. Furthermore, *in vivo* verification was executed on the xenografted mice. We hope our results can establish a theoretical basic for the therapy of thyroid cancer in the clinical practice.

## 2. Materials and Methods

### 2.1. Cell Culture

Human thyroid cancer cell lines IHH-4 (JCRB1079) and CAL-62 (CL-0618) were bought from JCRB Cell Bank (Tokyo, Japan) and Procell (Wuhan, China), respectively. Both of cells were cultured in the DMEM medium (PM150210, Procell) with 10% fetal bovine serum (FBS, 164210–500, Procell) and 1% streptomycin-penicillin (P/S, PB180120, Procell) at 37°C with 5% CO_2_.

### 2.2. Cell Counting Kit-8 (CCK-8) Assay

IHH-4 and CAL-62 cells with an inoculation density of 1 × 10^5^ cells/well were sowed into 96-well plates and incubated at 37°C with 5% CO_2_ overnight. After cells were administrated with diverse concentrations (0, 0.3, 0.6, 1.25, 2.5, 5, 10, 20, 40, and 80 *μ*M) of neferine for 96 h, 10 *μ*l CCK-8 reagent (Dojindo Laboratories, Kumamoto, Japan) was added into each well and then incubated at 37°C for 2 h. The microplate reader (Thermo Fisher Scientific, Waltham, MA, USA) was applied to record the absorbance at 450 nm. In addition, to determine the effect of neferine on the proliferation of IHH-4 and CAL-62 cells, both cells were inoculated into 96-well plates and maintained at 37°C in 5% CO_2_. After being cultured for 1 d, cell density was normalized and then incubated with 5 and 10 *μ*M neferine for further 48, 72, and 96 h, and the proliferation of cells was assessed by CCK-8. Neferine was bought from MedChemExpress (Monmouth Junction, NJ, USA, Cat No.: HY-N0441, CAS No.: 2292-16-2, purity: 99.92%) and dissolved in 0.1% dimethylsulfoxide (DMSO, Beyotime, Shanghai, China).

### 2.3. Flow Cytometry Analysis

The apoptosis of IHH-4 and CAL-62 cells was examined by the flow cytometry assay [20]. In brief, IHH-4 and CAL-62 cells were sowed into 24-well plates with a density of 2.5 × 10^5^ cells/well and cultured overnight at 37°C with 5% CO_2_. After being treated with 5 and 10 *μ*M neferine, both cells were gathered, rinsed with phosphate buffer saline (PBS) (Solarbio, Beijing, China), resuspended with 0.5 mL bind buffer, and stained with 5 *μ*L Annexin V/FITC (Thermo Fisher Scientific) and 5 *μ*L propidium iodide (PI) (Thermo Fisher Scientific) at room temperature for 15 min. The cells apoptosis was evaluated on FACScan flow cytometry with CellQuest software (BD Biosciences, NJ, USA).

### 2.4. Western Blot

Western blot assays were conducted as previous reports [21]. In brief, cells or tumor tissues were lysed with RIPA lysis buffer (Beyotime), and the concentration of total protein was calculated with the BCA kit (Thermo Fisher Scientific) based on the operation manual. 20 *μ*g protein samples were dissolved with 10% SDS-PAGE and then electrically transferred onto PVDF membranes (Millipore, Billerica, MA, USA) for the conventional processes of the western blot experiment. After being rinsed three times, the membrane was treated with goat anti-rabbit IgG H&L (HRP) (1 : 50000, ab205718, Abcam, Cambridge, UK) for 1 h at 37°C. The bands were visualized with the DAB kit (Sigma, St. Louis, MO, USA), and the gray value was measured by QUANTITY ONE software (Bio‐Rad, Hercules, CA, USA). The primary antibodies applied in the present study were anti-Ki67 (1 : 1000, ab16667), anti-Bax (1 : 500, ab53154), anti-Bcl-2 (1 : 2000, ab196495), anti-cleaved caspase-3 (1 : 500, ab2302), anti-VEGF (1 : 1000, ab46154), anti-E-cadherin (1 : 1000, ab40772), anti-N-cadherin (1 : 1000, ab18203), anti-Vimentin (1 : 1000, ab45939), anti-SLC7A11 (1 : 1000, ab216876), anti-GPX4 (1 : 1000, ab231174), anti-Nrf2 (1 : 1000, ab92946), anti-HO-1 (1 : 2000, ab13243), anti-NQO1 (1 : 3000, ab227520), anti-Lamin B1 (1 : 1000, ab16048), and anti-*β*-actin (1 : 5000, ab8227, all from Abcam).

### 2.5. Transwell Assay

The cell invasion was analyzed by the transwell assay based on the previous description [22]. In brief, IHH-4 and CAL-62 cell suspension with about 5 × 10^4^ cells was filled into the upper chambers containing the FBS-free medium and matrix, whereas the lower chamber was supplemented with the medium including 10% FBS. After being maintained for 24 h, both cells in the lower chamber were immobilized with 4% paraformaldehyde, stained with 0.1% crystal violet, and imaged with a microscope (Olympus, Tokyo, Japan).

### 2.6. Iron Assay

IHH-4 and CAL-62 cells with an inoculation density of 1 × 10^5^ cells/well were seeded into 96-well plates and incubated for 24 h at 37°C with 5% CO_2_. Subsequently, both cells were introduced with 5 *μ*M neferine, 10 *μ*M neferine, 5 *μ*M Ferrostatin-1 (Fer-1, the inhibitor of ferroptosis), 5 *μ*M neferine plus 5 *μ*M Fer-1, and 10 *μ*M neferine plus 5 *μ*M Fer-1 for 24 h. The relative iron level of cells was measured by an iron assay kit (Abcam, Cambridge, UK) in line with the handling instruction.

### 2.7. Determination of the ROS Level

Following administration with 5 *μ*M neferine, 10 *μ*M neferine, 5 *μ*M Fer-1, 5 *μ*M neferine plus 5 *μ*M Fer-1, and 10 *μ*M neferine plus 5 *μ*M Fer-1 for 24 h, IHH-4 and CAL-62 cells were washed and treated with 5-(and-6)-chloromethyl-2-,7-dichlorofluorescin diacetate (DCHF-DA) for 30 min at 37°C in the dark. Then, the relative fluorescence intensities of IHH-4 and CAL-62 cells were analyzed by FACScan flow cytometry (BD Biosciences).

### 2.8. Measurement of Glutathione (GSH) Level

After IHH-4 and CAL-62 cells were hatched with 5 *μ*M neferine, 10 *μ*M neferine, 5 *μ*M Fer-1, 5 *μ*M neferine plus 5 *μ*M Fer-1, and 10 *μ*M neferine plus 5 *μ*M Fer-1 for 24 h, the level of GSH was detected with the total glutathione/oxidized glutathione assay kit (A061-2-1, Nanjing Jiancheng Bioengineering Institute, Nanjing, China) based on the instruction description. The absorbance was read at 405 nm with a microplate reader (Thermo Fisher Scientific).

### 2.9. Cell Transfection

According to the previous study [21], transfection assays were carried out as follows: the Nrf2 sequence was cloned and inserted into pcDNA3 plasmid to overexpress the Nrf2 (pcDNA-Nrf2). The pcDNA-Nrf2 plasmid or pcDNA3 empty plasmid was then transfected into IHH-4 and CAL-62 cells with lipofectamine 3000 (Invitrogen, Carlsbad, CA, USA). Following transfection for 48 h, both cells were collected for further determinations.

### 2.10. *In Vivo* Analysis

All animal experiments were determined by the Animal Research Ethics Committee of the First Hospital of Shanxi Medical University. 4-week-old BALB/*c* nude mice were bought from Vital River (Beijing, China) and housed in a temperature-controlled SPF animal room with a 12 h cycle of light-dark. Ten mice were subcutaneously inoculated with a total of 2 × 10^6^ of IHH-4 cells [23, 24] and then assigned into two groups randomly: control group and neferine 20 mg/kg group. Mice in the neferine 20 mg/kg group were intraperitoneally administrated with 20 mg/kg neferine [25, 26], while mice in the control group were obtained with the same amount of DMSO. The tumor volume and mice body weight were monitored every five days. After sequential monitoring for 30 days, mice were sacrificed with an intraperitoneal injection of sodium pentobarbital (100 mg/kg), and the tumors samples were extracted, weighed, and stored for the following examination. Tumor volume was calculated according to the following formula: volume = 1/2 × length × width [2].

### 2.11. TUNEL Assay

The TUNEL assay was conducted with an *in situ* cell death detection kit (Roche, Budapest, Hungary) based on the operational instruction. The tumor tissues were immobilized in 4% formaldehyde solution and then embedded with paraffin. Tumor tissues were cut into 5 *μ*m thickness sections, dehydrated with the graded concentrations of ethanol, and cleared in xylene. Sections were then incubated with proteinase K for 20 min at 37°C and sealed with 3% H_2_O_2_ for 10 min followed by immobilization with 4% paraformaldehyde. Subsequently, sections were treated with the TUNEL reaction mixture at 37°C for 1 h. After the biotinylated antibody was incubated with sections, TUNEL positive cell number among the total cell number was counted.

### 2.12. Immunohistochemistry (IHC)

As per previous description [23], tumor tissues were removed, immobilized in 4% formaldehyde, dehydrated, embedded, and cut into sections. Sections were repaired with 10 mM sodium citrate buffer (pH 6.0, Beyotime) for 15 min at 94°C and cooled to room temperature. Subsequently, sections were continuously sealed with1% bovine serum albumin (BSA, Beyotime) for 30 min and treated with primary antibodies (VEGF, 1 : 1000, ab51745, Abcam) and biotinylated secondary antibody. Next, after being rinsed with PBS for three times, sections were restained with hematoxylin and imaged with a light microscope (Olympus).

### 2.13. Statistical Analysis

Data were displayed as the mean ± standard deviation (SD) and analyzed by SPSS 20.0 software (IBM, Armonk, New York, USA). Statistical differences between the two groups were tested by Student's *t*-test, whereas the differences among multiple groups were detected with one-way analysis of variance (ANOVA) followed by *post hoc* Bonferroni test. *P* < 0.05 was considered significant difference.

## 3. Results

### 3.1. Neferine Inhibits the Growth of Thyroid Cancer Cells

**Figure 1:**
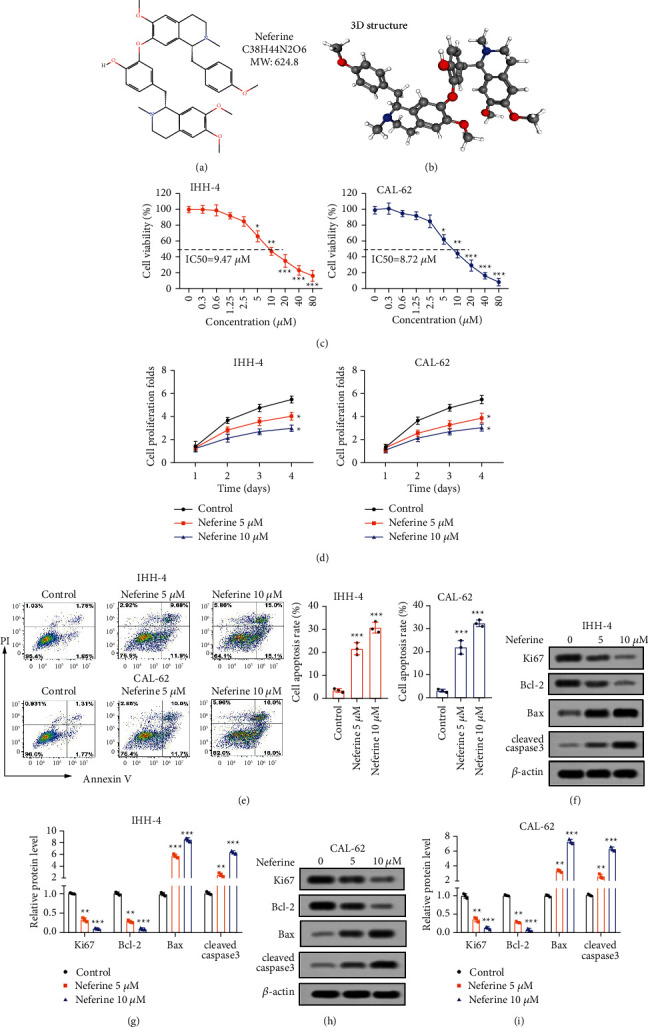
Neferine suppressed the proliferation and enhanced apoptosis of thyroid cancer cells. (a, b) The 2D and 3D structure of neferine. (c) The viability of IHH-4 and CAL-62 cells was detected by the CCK-8 assay after both cells were treated with a series of neferine, including 0, 0.3, 0.6, 1.25, 2.5, 5, 10, 20, 40, and 80 *μ*M for 96 h ^*∗*^*P* < 0.05, ^*∗∗*^*P* < 0.01, and ^*∗∗∗*^*P* < 0.001 vs. 0 *μ*M. (d) The viability of IHH-4 and CAL-62 cells was examined by the CCK-8 assay after both cells were administrated with 5 and 10 *μ*M neferine for 24, 48, 72, and 96 h ^*∗*^*P* < 0.05 vs. control. (e) The apoptosis rate was determined by flow cytometry after IHH-4 and CAL-62 cells were incubated with 5 and 10 *μ*M neferine for 96 h ^*∗∗∗*^*P* < 0.001*vs.* Control. (f–i) The relative protein expression of Ki67, Bax, Bcl-2, and cleaved caspase-3 was measured by western blot assays. The data were expressed after being normalized to *β*-actin. ^*∗∗*^*P* < 0.01 and ^*∗∗∗*^*P* < 0.001*vs.* control. All assays were performed in triplicate.

To determine the effect of neferine (Figures 1(a) and 1(b)) on the progress of thyroid cancer, IHH-4 and CAL-62 cells were first stimulated with a variety of neferine, including 0, 0.3, 0.6, 1.25, 2.5, 5, 10, 20, 40, and 80 *μ*M for 96 h. CCK-8 results showed that neferine repressed the cell viability of both IHH-4 and CAL-62 cells in a dose-dependent way, among which neferine ranged from 5 *μ*M to 80 *μ*M notably suppressing the cell viability of both IHH-4 and CAL-62 cells (Figure 1(c)). Meanwhile, the IC50 value of neferine on IHH-4 and CAL-62 cells was 9.47 and 8.72 *μ*M, respectively (Figure 1(c)). Thus, two concentrations of neferine, including 5 and 10 *μ*M, were selected for subsequent assays. Results from Figure 1(d) figured that both 5 and 10 *μ*M neferine induced a prominent decrease in viability of both IHH-4 and CAL-62 cells, among which both 5 and 10 *μ*M neferine observably reduced the viability of both IHH-4 and CAL-62 cells at 96 h. In addition, both 5 and 10 *μ*M neferine markedly increased the apoptosis rate of IHH-4 and CAL-62 cells (Figure 1(e)), accompanied with a remarkable diminishment of the relative protein level of Bcl-2 and an obvious enhancement of the relative protein expression of Bax and cleaved caspase-3 (Figures 1(f)–1(i)). Besides, both 5 and 10 *μ*M neferine also markedly declined the relative protein expression of Ki67 of IHH-4 and CAL-62 cells (Figures 1(f)–1(i)). Therefore, these results indicated that neferine inhibited the growth and promoted apoptosis of thyroid cancer cells.

### 3.2. Neferine Suppressed Thyroid Cancer Cell Invasion and EMT

**Figure 2:**
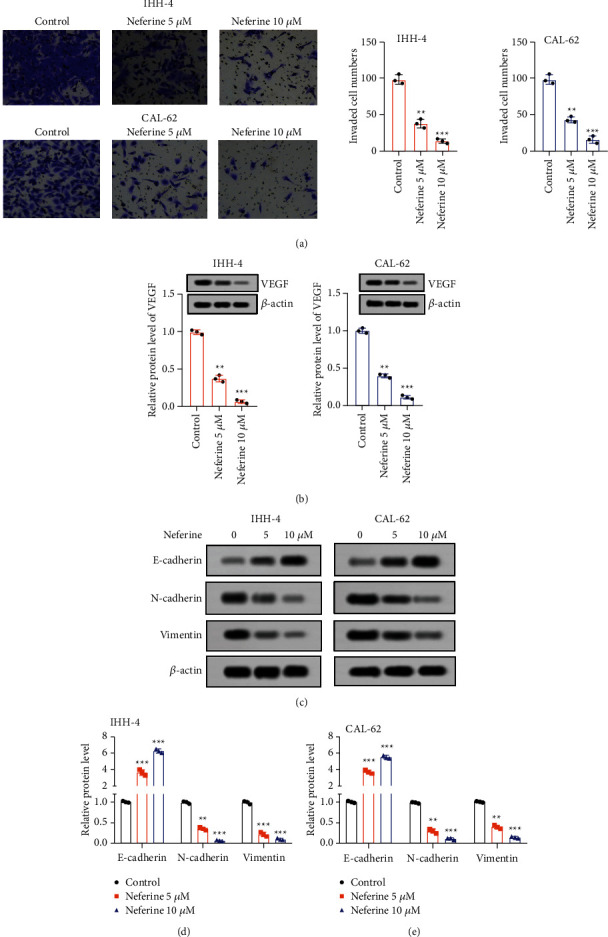
Neferine restrained the invasion and EMT of thyroid cancer cells. IHH-4 and CAL-62 cells were exposed with 5 and 10 *μ*M neferine for 96 h. (a) The invaded cell numbers were detected by transwell assays. (b–e) The relative protein expression of VEGF, E-cadherin, N-cadherin, and vimentin was analyzed by the western blot assay. The data were expressed after being normalized to *β*-actin. ^*∗∗*^*P* < 0.01 and ^*∗∗∗*^*P* < 0.001*vs.* control. All assays were performed in triplicate.

Transwell results exhibited that both 5 and 10 *μ*M neferine expectedly declined the invaded cell numbers of IHH-4 and CAL-62 cells prominently, indicating an extremely repressive effect of neferine on the invasion of thyroid cancer cells (Figure 2(a)). Also, both 5 and 10 *μ*M neferine induced a noteworthy reduction of the relative protein expression of VEGF in IHH-4 and CAL-62 cells (Figure 2(b)). Moreover, both 5 and 10 *μ*M neferine memorably increased the relative protein expression of E-cadherin, while notably declined the relative protein level of N-cadherin and vimentin of IHH-4 and CAL-62 cells, suggesting that neferine overtly hindered the EMT of thyroid cancer cells (Figures 2(c)–2(e)). Thus, neferine suppressed the invasion and EMT of thyroid cancer cells.

### 3.3. Neferine Induces Ferroptosis in Thyroid Cancer Cells

**Figure 3:**
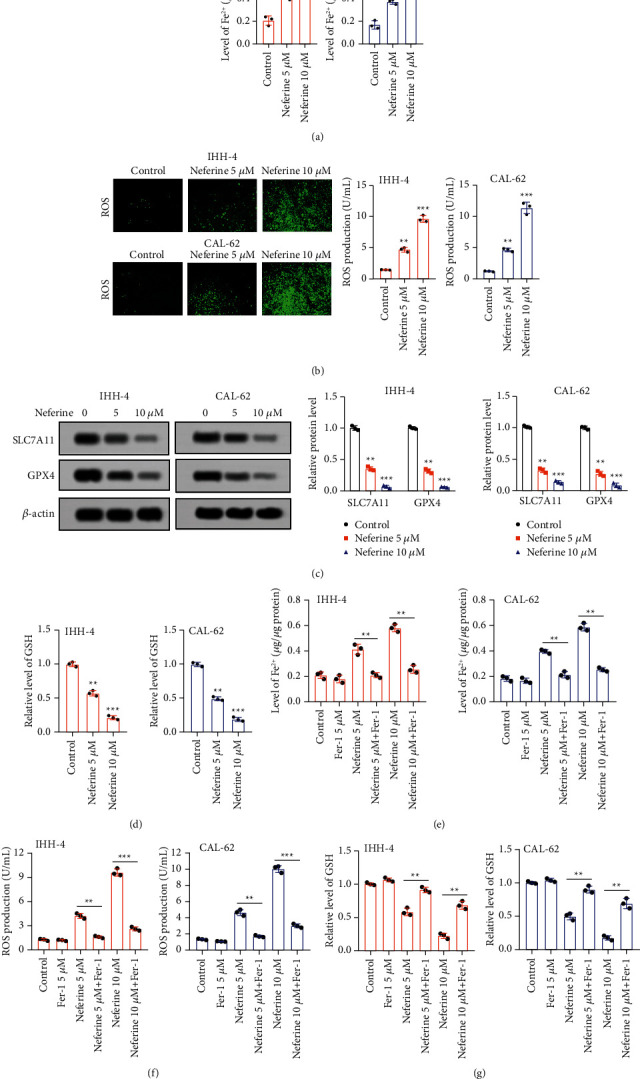
Neferine induced the activation of ferroptosis in thyroid cancer cells. (a) The relative iron level was measured by an iron assay kit. ^*∗∗*^*P* < 0.01 and ^*∗∗∗*^*P* < 0.001*vs.* control. (b) The ROS level was detected by flow cytometry after IHH-4 and CAL-62 cells were treated with DCHF-DA. ^*∗∗*^*P* < 0.01 and ^*∗∗∗*^*P* < 0.001*vs.* control. (c) The relative protein expressions of both SLC7A11 and GPX4 were determined by the western blot. The data were expressed after being normalized to *β*-actin. ^*∗∗*^*P* < 0.01 and ^*∗∗∗*^*P* < 0.001*vs.* control. (d) The levels of GSH were quantified by using the commercial kit. ^*∗∗*^*P* < 0.01 and ^*∗∗∗*^*P* < 0.001*vs.* control. (e–g) After IHH-4 and CAL-62 cells were stimulated with Fer-1, neferine or their combination, the level of Fe^2+^, ROS production, and the relative level of GSH were assessed by using the iron assay kit, flow cytometry, and commercial kit, respectively. ^*∗∗*^*P* < 0.01 and ^*∗∗∗*^*P* < 0.001. All assays were performed in triplicate.

To investigate the effect of neferine on ferroptosis in thyroid cancer cells, the level of Fe^2+^ was first measured after IHH-4 and CAL-62 cells were hatched with 5 and 10 *μ*M neferine. The results revealed that both 5 and 10 *μ*M neferine significantly elevated the level of Fe^2+^ of IHH-4 and CAL-62 cells (Figure 3(a)). Consistently, 5 and 10 *μ*M neferine also induced a remarkable enhancement of ROS production of IHH-4 and CAL-62 cells (Figure 3(b)). On the contrary, the relative protein expressions of SLC7A11 and GPX4 were notably reduced after IHH-4 and CAL-62 cells were hatched with 5 and 10 *μ*M neferine (Figure 3(c)). A prominent decrease of the relative GSH level was also observed in IHH-4 and CAL-62 cells induced with 5 and 10 *μ*M neferine (Figure 3(d)). Moreover, to further validate the stimulative effect of neferine on ferroptosis in thyroid cancer cells, IHH-4 and CAL-62 cells were treated with Fer-1, a recognized inhibitor of ferroptosis. The results revealed that Fer-1 administration significantly reversed the neferine-induced changes in the level of Fe^2+^, ROS production, and the relative level of GSH of IHH-4 and CAL-62 cells (Figures 3(e)–3(g)). Hence, neferine activated ferroptosis in thyroid cancer cells.

The anticancer effect of neferine on thyroid cancer was involved in the Nrf2/HO-1/NQO1 pathway.

**Figure 4:**
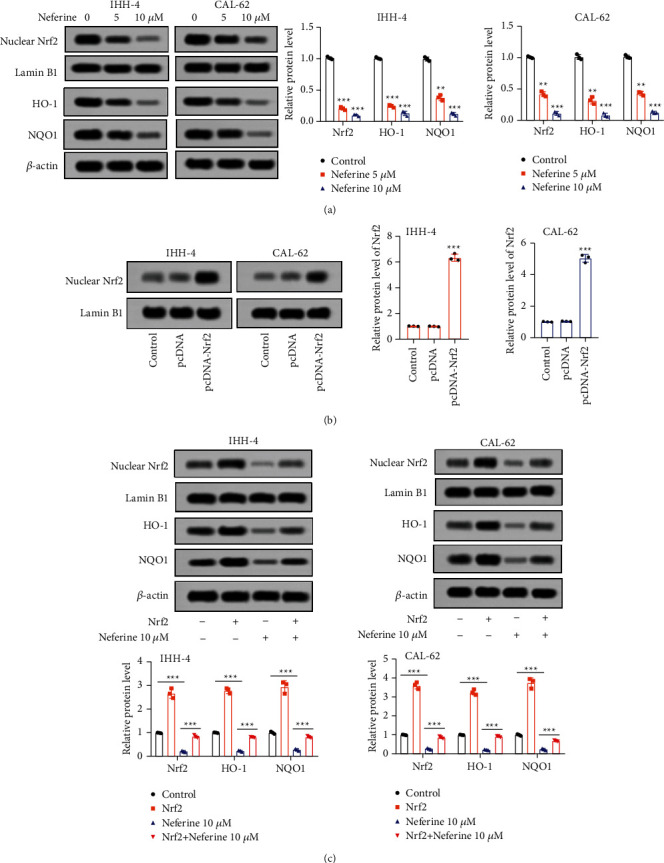
Neferine downregulated the Nrf2/HO-1/NQO1 signaling in thyroid cancer cells. (a) The relative protein expressions of Nrf2, HO-1, and NQO1 were detected after IHH-4 and CAL-62 cells were exposed with both 5 and 10 *μ*M neferine for 96 h ^*∗∗*^*P* < 0.01 and ^*∗∗∗*^*P* < 0.001*vs.* control. (b) Nrf2 sequences were cloned and inserted into the pcDNA3 plasmid to overexpress Nrf2 via the transfection into IHH-4 and CAL-62 cells with lipofectamine 3000. ^*∗∗∗*^*P* < 0.001*vs.* control. (c) The relative protein expressions of Nrf2, HO-1, and NQO1 were determined after IHH-4 and CAL-62 cells were treated with Nrf2, neferine, or their combination. The data were expressed after being normalized to lamin B1 or *β*-actin. ^*∗∗∗*^*P* < 0.001. All assays were performed in triplicate.

**Figure 5:**
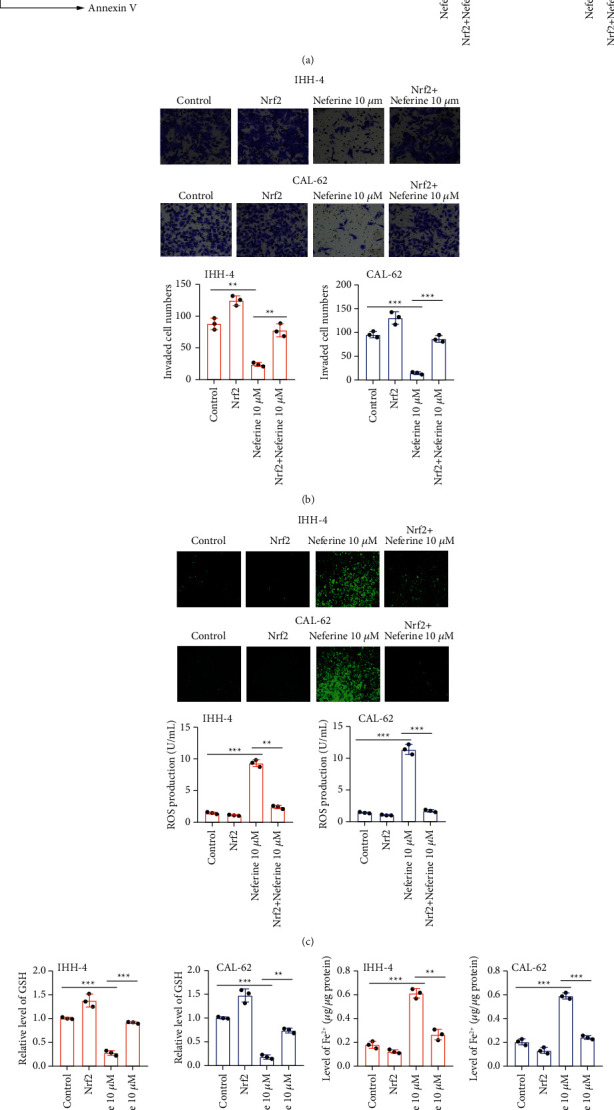
The anticancer effect of neferine on thyroid cancer was associated with the Nrf2/HO-1/NQO1 pathway. IHH-4 and CAL-62 cells were treated with Nrf2, neferine, or their combination. (a) The apoptosis rate was examined by flow cytometry. (b) The invaded cell numbers were determined by transwell assays. (c) The ROS level was measured by flow cytometry after IHH-4 and CAL-62 cells were hatched with DCHF-DA. (d) The levels of GSH were analyzed by using the commercial kit. (e) The relative iron level was detected by an iron assay kit. ^*∗∗*^*P* < 0.01 and ^*∗∗∗*^*P* < 0.001. All assays were performed in triplicate.

To determine the underlying mechanism of neferine on thyroid cancer, the relative protein expressions associated with the Nrf2/HO-1/NQO1 pathway were detected by the western blot. The relative protein expressions of Nrf2, HO-1, and NQO1 were all observably downregulated after IHH-4 and CAL-62 cells were exposed with both 5 and 10 *μ*M neferine (Figure 4(a)). To further verify the direct connection between the anticancer effect of neferine and the Nrf2/HO-1/NQO1 pathway, Nrf2 was overexpressed in IHH-4 and CAL-62 cells (Figure 4(b)). As expected, overexpression of Nrf2 markedly elevated the relative protein expressions of Nrf2, HO-1, and NQO1 of IHH-4 and CAL-62 cells (Figure 4(c)). Meanwhile, upregulation of Nrf2 also significantly partly rescued the neferine-induced relative protein level of Nrf2, HO-1, NQO1 of IHH-4 and CAL-62 cells (Figure 4(c)). Furthermore, Nrf2 overexpression alone prominently declined the apoptosis rate, and Nrf2 overexpression also notably antagonized the neferine-induced apoptosis rate of IHH-4 and CAL-62 cells (Figure 5(a)), while an inverse tendency was discovered in the invaded cell numbers (Figure 5(b)). More importantly, Nrf2 overexpression neutralized the neferine-induced ROS production, the relative level of GSH, and the level of Fe^2+^ of IHH-4 and CAL-62 cells (Figures 5(c)–5(e)). Nrf2 overexpression alone observably reduced ROS production and the level of Fe^2+^, whereas enhanced the relative level of GSH of IHH-4 and CAL-62 cells (Figures 5(c)–5(e)). Altogether, these data illustrated that the anticancer effect of neferine on thyroid cancer was involved in the Nrf2/HO-1/NQO1 pathway.

### 3.4. Neferine Inhibits Tumorigenesis and Induces Ferroptosis in Nude Mice

**Figure 6:**
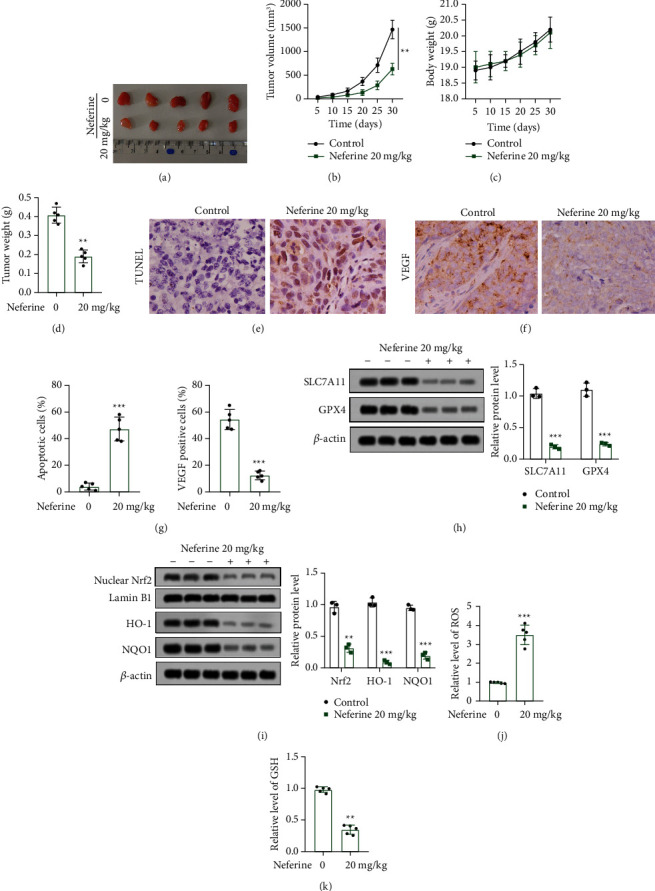
Neferine impeded tumorigenesis and induced ferroptosis *in vivo* involving in the Nrf2/HO-1/NQO1 pathway. (a) Representative images of tumors from nude mice in the control group and neferine 20 mg/kg group. (b, c) The tumor volumes and mice body weight were monitored every five days. ^*∗∗*^*P* < 0.01. (d) Tumor samples were weighted after mice were administrated with 30 days. ^*∗∗*^*P* < 0.01*vs.* 0 mg/kg neferine. (e–g) The apoptosis and VEGF expression were detected by TUNEL and IHC, respectively. ^*∗∗∗*^*P* < 0.001*vs.* 0 mg/kg neferine. (h) The relative protein expressions of SLC7A11 and GPX4 were examined by the western blot. The data were expressed after being normalized to *β*-actin. ^*∗∗∗*^*P* < 0.001*vs.* control. (i) The relative protein expressions of Nrf2, HO-1, and NQO1 were assessed by the western blot. The data were expressed after being normalized to lamin B1 or *β*-actin. ^*∗∗*^*P* < 0.01 and ^*∗∗∗*^*P* < 0.001*vs.* control. (j, k) The levels of ROS (j) and GSH (k) were measured using commercial kits. ^*∗∗*^*P* < 0.01 and ^*∗∗∗*^*P* < 0.001*vs.* 0 mg/kg neferine. All assays were performed in triplicate.

In addition, the inhibitory effect and relevant mechanisms of neferine on thyroid cancer were validated *in vivo*. BALB/*c* nude mice were subcutaneously received with a total of 2 × 10^6^ of IHH-4 cells and then intraperitoneally injected with 20 mg/kg neferine. Neferine treatment significantly reduced the tumor volume and weight, though no statistical difference was observed in mice body weight between the control group and the neferine 20 mg/kg group (Figures 6(a)–6(d)). Similar to the *in vitro* results, neferine administration notably promoted apoptosis and inhibited the VEGF level in xenografted mice (Figures 6(e)–6(g)). Also, neferine introduction significantly decreased the relative protein expression of SLC7A11, GPX4, Nrf2, HO-1, and NQO1 but increased the relative level of ROS (Figures 6(h)–6(j)). Besides, the neferine injection markedly downregulated the relative level of GSH (Figures 6(h)–6(k)). Altogether, these data expounded that neferine suppressed tumorigenesis and induced ferroptosis in nude mice, which was associated with the Nrf2/HO-1/NQO1 pathway.

## 4. Discussion

Thyroid cancer is the most prevalent endocrine malignancy with an increasing incidence in the past few decades due to various contributors, such as immune infiltration [27, 28]. Neferine has a variety of pharmacological effects, and thus, it has been applied in diverse disease models, including tumors [16]. However, the detailed effect and mechanism of neferine on thyroid cancer are still unclear. In the current study, neferine suppressed the cell viability of both IHH-4 and CAL-62 cells both in a dose-dependent manner and in a time-dependent fashion. In addition, neferine promoted apoptosis, but it inhibited invasion, angiogenesis, and EMT of IHH-4 and CAL-62 cells. Moreover, neferine activated ferroptosis in thyroid cancer cells. Notably, it was revealed that the Nrf2/HO-1/NQO1 pathway was strongly associated with the effect of neferine on the modulation of thyroid cancer. Furthermore, these outcomes were validated in xenografted mice. Therefore, based on these results, we concluded that neferine exerted an antitumor effect and ferroptosis-inducing effect on thyroid cancer through Nrf2/HO-1/NQO1 inhibition.

A series of hallmarks have been identified in tumors, such as proliferation, apoptosis, invasion, angiogenesis, and EMT [29], thus agents targeting these characteristics are potential candidates for the treatment of cancers. Neferine has been demonstrated to regulate the progresses of a variety of tumors. The inhibitory effect of neferine on the growth and migration has been discussed in prostate cancer [17, 30], cervical cancer [19], and retinoblastoma [31], and the encouraging role of neferine in apoptosis has also been reported in prostate cancer [32], renal cancer [33], and colon cancer [34]. In line with these studies, our results also showed that neferine suppressed the cell viability of both IHH-4 and CAL-62 cells both in a dose-dependent manner and in a time-dependent fashion. Besides, neferine also declined the relative protein expression of Ki67 of IHH-4 and CAL-62 cells. Ki67 is a positive marker of the proliferation of tumors [35]. Thus, neferine inhibited the growth of thyroid cancer cells, which was also confirmed *in vivo*. Meanwhile, neferine increased the apoptosis rate of IHH-4 and CAL-62 cells, accompanied with a remarkable diminishment of the Bax expression and an obvious enhancement of the Bcl-2 and cleaved caspase-3 levels. Bax, Bcl-2, and cleaved-caspase-3 are pivotal proteins of apoptosis [36].Therein, proapoptosis protein Bax and antiapoptosis protein Bcl-2 are two vital members of Bcl-2 family, which modulate the apoptosis [37]. Cleaved-caspase-3 is the activation form of caspase-3 that regulated the various phases in the apoptotic pathway [38]. In addition, neferine reduced the invaded cell numbers, indicating that neferine suppressed the invasion of thyroid cancer cells. Meanwhile, neferine decreased the VEGF expression of IHH-4 and CAL-62 cells. VEGFs are important signaling of angiogenesis, which is essential for tumor growth and enables metastasis [39]. Thus, neferine might also inhibit the angiogenesis of thyroid cancer cells; however, the tube formation assay and other marker detection are needed in the subsequent assays. Furthermore, neferine increased the relative protein expression of E-cadherin, while decreased the relative protein expression of N-cadherin and vimentin of IHH-4 and CAL-62 cells, suggesting that neferine hindered the EMT of thyroid cancer cells. Diminishment of E-cadherin leads to the reduction of adhesion and the increase of invasion and migration, whereas inverse effects are observed in N-cadherin and vimentin [40–42]. Hence, upregulation of N-cadherin and vimentin with downregulation of E-cadherin are the hallmark of EMT [43]. In all, these results indicated that neferine inhibited the proliferation, invasion, angiogenesis, and EMT but promoted apoptosis of thyroid cancer both *in vitro* and *in vivo*.

Ferroptosis is an emerging feature of tumor including thyroid cancer, and ferroptosis activation is an underlying option for the treatment of thyroid cancer. CircKIF4A dampens ferroptosis to enhance the malignant development of papillary thyroid cancer [15]. Vitamin C can be the underlying strategy for anaplastic thyroid cancer therapy through the induction of ferroptosis [14]. Here, *in vitro* and *in vivo* results from the present study exhibited that neferine elevated the level of Fe^2+^ and ROS production, but decreased the relative GSH level and the relative protein expressions of SLC7A11 and GPX4. Iron plays a key role in the growth of cells, especially for tumor cells, whose demand is higher than normal cells [44]. ROS are strongly related in the various progresses of tumor cells. Notably, the deposition of ROS can directly cause ferroptosis generated by mitochondria [45]. GPX4 and SLC7A11 are two critical ferroptosis-related proteins. Among them, GPX4, a member of the glutathione peroxidase family, plays a vital role in the ferroptosis resistance [46]. Thus, inhibition of GPX4 induces ferroptosis [47]. SLC7A11 is a composition of the cystine/glutamate antiporter that is principally involved in the transportation of extracellular cystine into cells in exchange for glutamate [48]. Inhibition of xCT has been demonstrated to impeded cystine uptake and has resulted in ferroptosis [49, 50]. Repression of GSH synthesis leading to ferroptosis is one of the essential ways of modulation and signaling of ferroptosis. GSH is a momentous intracellular antioxidant that protects cells from oxidative injury. Aberrant GSH synthesis can cause the accumulation of lipid peroxidation, which results in ferroptosis [51]. Thus, our results also suggested that neferine inhibited ferroptosis of thyroid cancer cells, which was further validated with the application of the ferroptosis inhibitor, Fer-1. Altogether, neferine activated ferroptosis in thyroid cancer cells.

Transcription factor Nrf-2 is an crucial regulator for a variety of cell biological processes, especially oxidative stress [52], which can modulate the expression of antioxidant enzymes and conjugation/detoxification enzymes, such as HO-1 and NQO-1 [53]. It has been revealed that both HO-1 and NQO-1 can neutralize the oxidative stress through enhancing the removal of ROS [54]. Activated HO-1 can prevent oxidative injury and regulate the infiltrating inflammatory cells, thus it is generally directly involved in the mobility and invasion of tumor cells [55]. On the other hand, NQO-1 can suppress carcinogenesis via stabilizing the P53 tumor suppressor [56]. Nrf2 signaling is strongly associated with papillary thyroid cancer [57], anaplastic thyroid cancer [58], and thyroid cancer [59]. Moreover, the regulative effect of neferine on Nrf2 signaling is also revealed in different disease models, such as benign prostate hyperplasia [60] and esophageal squamous cell carcinoma [61]. In the present study, neferine downregulated the relative protein expressions of Nrf2, HO-1, and NQO1 both *in vitro* and *in vivo*, which could be partly rescued by overexpression of Nrf2 *in vitro*. In addition, upregulation of Nrf2 also partly reversed the neferine-induced apoptosis and invasion of IHH-4 and CAL-62 cells. More importantly, Nrf2 overexpression neutralized the neferine-induced ROS production, the relative level of GSH, and the level of Fe^2+^ of IHH-4 and CAL-62 cells. Song et al. [62] summarizes the mechanism of Nrf2 modulating ferroptosis in neurodegenerative diseases. Wu et al. [63] shows that the high-mobility group box-1 (HMGB1) modulates ferroptosis induced by high glucose in mesangial cells through the Nrf2 pathway. Therefore, these outcomes elaborated that the anticancer effect of neferine on thyroid cancer was associated with the Nrf2/HO-1/NQO1 pathway.

In conclusion, the present study elucidated that neferine exerted an antitumor effect and ferroptosis-inducing effect on thyroid cancer, which was involved in the inhibition of the Nrf2/HO-1/NQO1 signaling pathway. In brief, our results offer a solid theoretical foundation for clinical development and therapy of thyroid cancer.

## Data Availability

All data, models, and code generated or used during the study appear in the submitted article.

## Additional Points

Thyroid cancer is the most prevalent endocrine malignancy with an increasing incidence in the past few decades, and thus, much exploration remains to contribute to the understanding and development of pathogenesis and treatment of thyroid cancer. Although neferine possesses various pharmacological activities, which have been applied in diverse disease models, including various tumors, the detailed effect and mechanism of neferine on the thyroid cancer are still unclear. The present study revealed that neferine exerted an antitumor effect and ferroptosis-inducing effect on thyroid cancer via inhibiting the Nrf2/HO-1/NQO1 pathway. As far as we know, this is the first time to report the effect of neferine on thyroid cancer. Besides, the effect of neferine on ferroptosis in thyroid cancer is first to be explored. In brief, our results offer a solid theoretical foundation for clinical development and therapy of thyroid cancer.
